# Foveal crack sign as a predictive biomarker for development of macular hole in fellow eyes of patients with full-thickness macular holes

**DOI:** 10.1038/s41598-020-77078-y

**Published:** 2020-11-16

**Authors:** Olga Furashova, Egbert Matthé

**Affiliations:** 1 Department of Ophthalmology, Klinikum Chemnitz gGmbH, Flemmingstrasse 2, 09116 Chemnitz, Germany; 2 Department of Ophthalmology, University Hospital Carl Gustav Carus, Technische Universität Dresden, Fetscherstrasse 74, 01307 Dresden, Germany

**Keywords:** Retinal diseases, Vision disorders

## Abstract

To investigate the prevalence and predictive value of the foveal crack sign (FCS) in fellow eyes of patients with full-thickness macular holes (FTMH) regarding future macular hole (MH) formation. In a retrospective observational case series, 113 fellow eyes of 113 patients with FTMH have been observed during a mean follow-up time of 21 months. According to baseline SD-OCT images, patients were divided into 4 separate groups: patients with FCS and vitreous adhesion, patients with FCS and vitreous detachment, patients without FCS with vitreous adhesion, patients without FCS with vitreous detachment. Progression rate to MH formation, predictive value of FCS and of vitreous interface status were calculated and compared across the four groups. FCS was observed in 19 of 113 fellow eyes (17%) of patients with FTMH, 10 of them with progression to MH during the mean follow up time of 21 months. 2 other eyes with progression to MH showed no FCS at baseline. Progression rate was shown to be 77% (10 of 13 eyes) in patients with FCS and vitreous adhesion, 0% (none of 6 eyes) in patients with FCS and vitreous detachment, 4% (2 of 48 eyes) in patients without FCS with vitreous adhesion, 0% (none of 46 eyes) in patients without FCS with vitreous detachment. FCS had sensitivity of 83.3% (95% CI 50.9–97.1%) and specificity of 91.1% (95% CI 83.3–95.6%) in predicting MH formation, positive predictive value of FCS was 52.6% (95% CI 29.5–74.8%) and negative predictive value 97.9% (95% CI 91.8–99.6%). Having simultaneously FCS and vitreous adhesion showed 83.3% (95% CI 50.9–97.1%) sensitivity and 97.1% (95% CI 91.1–99.2%) specificity in predicting macular hole formation; positive predictive value was 76.9% (95% CI 46.0–93.8%) and negative predictive value was 98.0% (95% CI 92.4–99.7%). Fellow eyes of patients with FTMH with foveal crack sign are at a very high risk (77%) of FTMH development, as long as posterior vitreous adhesion is present.

## Introduction

Patients with FTMH in one eye are at higher risk of developing the same condition in the fellow eye. The risk of the involvement of the second eye varies in most studies between 0 and 31%^1–3^. Lewis et al. found a 19% incidence of bilaterality at 48 months of follow up in a very large cohort of patients^4^. In a 5-year prospective natural study of Ezra et al., the incidence of idiopathic FTMH in the fellow eyes was as high as 10%^5^. Focusing on diagnostic biomarker preceding macular hole formation would be helpful to better monitor these eyes at higher risk.

Kumagi et al. showed that vitreoretinal interface seems to be altered in *both* eyes of patients with unilateral FTMH^6^. The rate of interface changes was the highest in fellow eyes of FTMH patients compared to other retinal pathologies. The role of this finding in the future MH development cannot be estimated, as this study was a cross-sectional study without follow-up.

Recently, Choi et al. underlined the importance of changes in the outer foveal region, innerfoveal cysts and vitreomacular adhesion or traction as possible risk factors for MH development^7^. The changes in the outer retina (“outer foveal defect”) described by Choi et al. were further analyzed in another recently published paper. Ishibashi et al. described a novel optical coherence tomography sign—foveal crack sign—which preceded macular hole formation after vitrectomy for rhegmatogenous retinal detachment in all 10 cases^8^. Another recent study of Scharf et al. observed the presence of a central hyperreflective line in the fovea on spectral-domain optical coherence tomography (SD-OCT) preceding FTMH and after resolution of FTMH after vitrectomy in approximately half of the cases^9^. They concluded that this hyperreflective line seen on SD-OCT images might represent an early diagnostic marker for FTMH development.

The purpose of the current study is to investigate the prevalence of the foveal crack sign in the fellow eyes of patients with FTMH and to determine its possible predictive value for future development of a macular hole.

## Results

In total, 113 fellow eyes from 113 patients with FTMH were included in this study. The demographic characteristics of the study participants are summarized in Table 1. There were no statistically significant differences across the four groups regarding age, sex, laterality, best corrected visual acuity (BCVA) and refractive error. The mean MH size of the fellow eyes was also similar across the groups. The mean follow up time in the whole cohort was 21 months (range 5–59 months). After post hoc Sidak’s test analysis, there remained a statistically significant difference in the follow up time between the patients without FCS and the group with FCS an posterior vitreous adhesion. Table 2 summarizes the SD-OCT characteristics of the different groups. Central foveal thickness was significantly higher in the groups with posterior vitreous adhesion (*p* = 0.006), which might be due to some kind of central vitreoretinal traction and therefore thickening of the central retina. Incidence of an epiretinal membrane (ERM) showed no statistically significant differences across the groups (*p* = 0.351). All ERM cases were mild, detected mostly in the papillomacular area without any visible tangential traction to the foveal area. Degenerative lamellar macular holes were detected significantly more often in both groups with posterior vitreous detachment (< 0.001*).

**Table 1:** Distribution of general characteristics of included patients across all the groups.

Characteristics	All eyes	FCS +		FCS −		*p* value
	N = 113	Vitreous adhesion group, N = 13	Vitreous detachment group, N = 6	Vitreous adhesion group, N = 48	Vitreous detachment group, N = 46	
Age, year (mean ± SD)	71 ± 7	68 ± 8	71 ± 5	71 ± 6	72 ± 8	0.412
**Sex (%)**						
Male	33 (29)	4 (31)	1 (17)	16 (33)	12 (26)	0.788
Female	80 (71)	9 (69)	5 (83)	32 (67)	34 (74)	
**Eye (%)**						
Right	48 (42)	10 (77)	3 (50)	18 (38)	17 (37)	0.364
Left	65 (58)	3 (23)	3 (50)	30 (62)	29 (63)	
BCVA, logMAR (mean ± SD)	0.08 ± 0.17	0.09 ± 0.10	0.12 ± 0.12	0.09 ± 0.19	0.06 ± 0.16	0.743
FTMH size in the fellow eye, µm (mean ± SD)	461 ± 168	471 ± 229	546 ± 189	432 ± 167	476 ± 146	0.347
Refractive error, D (mean ± SD)	0.61 ± 1.62	0.09 ± 1.51	0.27 ± 2.06	1.10 ± 1.50	0.29 ± 1.64	0.451
Follow up time, months (mean ± SD)	14 ± 10	24 ± 17	15 ± 11	13 ± 10	12 ± 7	0.003*

*BCVA* best corrected visual acuity, *logMAR* logarithm of the minimal angle of resolution, *FTMH* full-thickness macular hole, *FCS*+ eyes with foveal crack sign; *FCS− *eyes without foveal crack sign, *SD* standard deviation, *D* diopters. Differences across the four groups were calculated using 1-way multivariate factorial ANOVA with post hoc Sidak’s test and the chi-square test according to the variables.

*Statistically significant.

**Table 2:** OCT-morphological characteristics and progression rate in the separate groups.

Characteristics	All eyes, N = 113	FCS +		FCS −		*p* value
		Vitreous adhesion group, N = 13	Vitreous detachment group, N = 6	Vitreous adhesion group, N = 48	Vitreous detachment group, N = 46	
CFT, µm (mean ± SD)	270 ± 28	274 ± 34	246 ± 14	278 ± 30	262 ± 22	0.006*
Epiretinal membrane (%)	17 (15)	4 (31)	1 (17)	5 (10)	7 (15)	0.351
Degenerative LMH (%)	17 (15)	1 (8)	3 (50)	0 (0)	13 (28)	< 0.001*
Foveal crack sign (%)	19 (17)	13 (100)	6 (100)	0 (0)	0 (0)	N/A
Progression to MH formation (%)	12 (11)	10 (77)	0 (0)	2 (4)	0 (0)	0.000*

Differences across the four groups were calculated using 1-way ANOVA with post hoc Sidak’s test and chi-square test according to the variables.

*CFT* central foveal thickness, *LMH* lamellar macular hole, *FTMH* full-thickness macular hole, *FCS*+ eyes with foveal crack sign, *FCS− *eyes without foveal crack sign, *SD* standard deviation, *N/A* not applicable.

*Statistically significant.

The foveal crack sign could be seen only in 19 of 113 fellow eyes (17%) at baseline. Progression to FTMH formation was observed in 12 eyes, 10 of them displayed FCS at baseline. In the other 9 cases with FCS at baseline, FCS resolved in 5 eyes till the end of the follow-up time and remained present in 4 cases.

**Figure 1:**
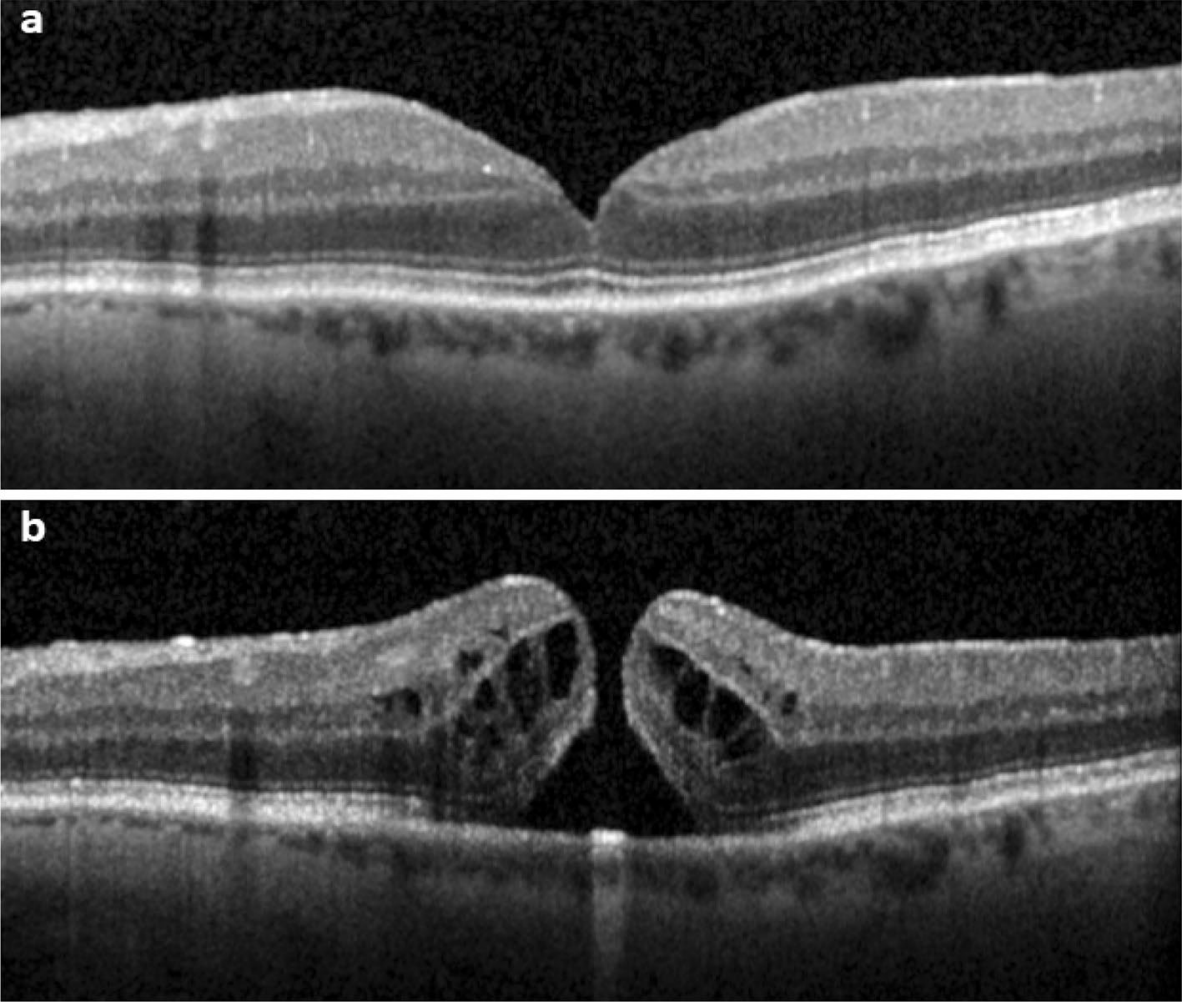
SD-OCT of the fovea (horizontal line scan) with the foveal crack sign preceding macular hole formation in a 76-year-old-man. **(a)** Foveal crack sign in the umbo of the left eye with mild vitreomacular traction. **(b)** Progression to full-thickness macular hole formation 9 months later.

**Figure 2:**
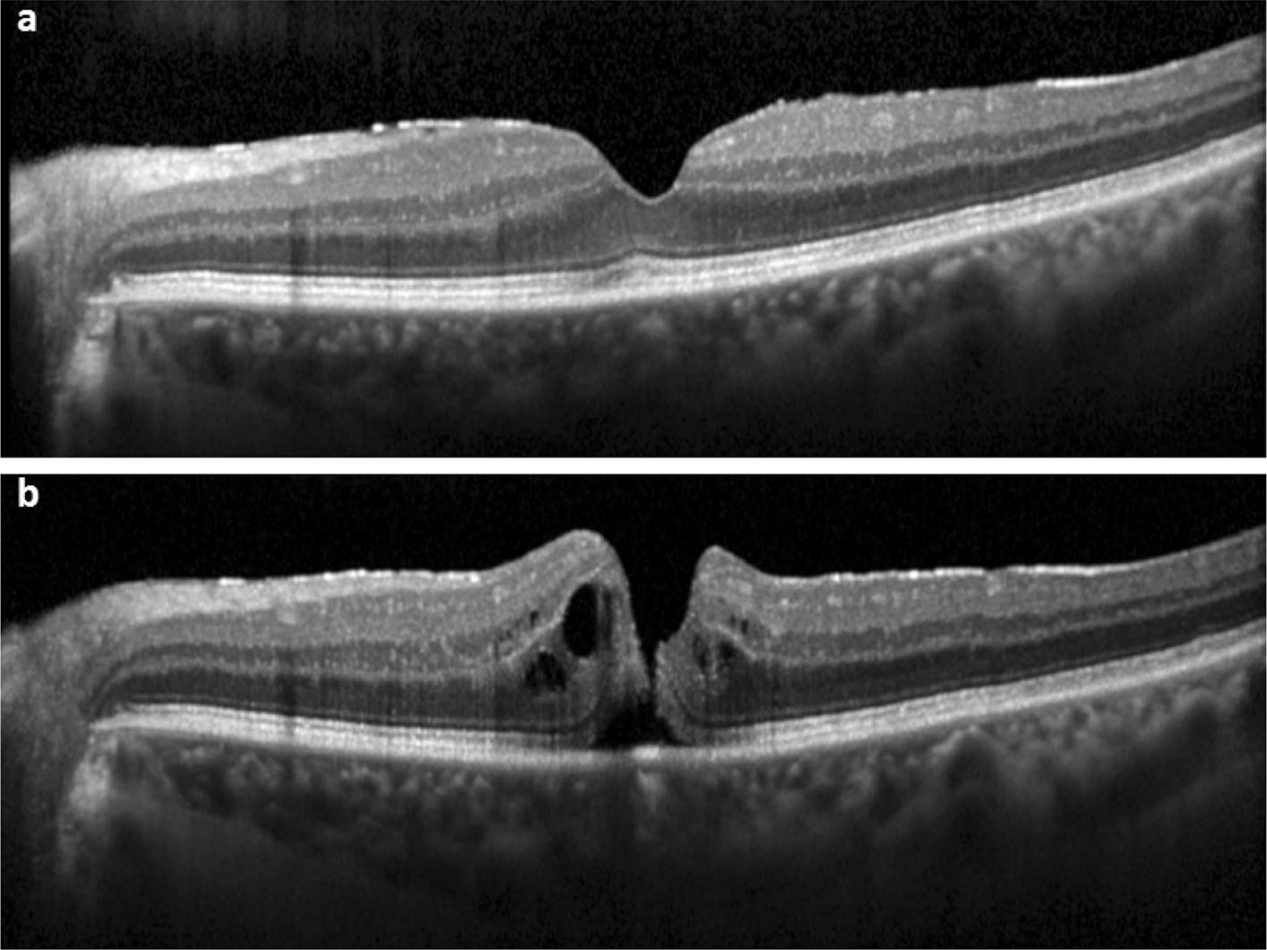
SD-OCT of the fovea (horizontal line scan) without the foveal crack sign preceding macular hole formation in a 72-year-old-woman. **(a)** Mild epiretinal membrane without foveal crack sign. **(b)** Progression to full-thickness macular hole formation 14 months later.

**Figure 3:**
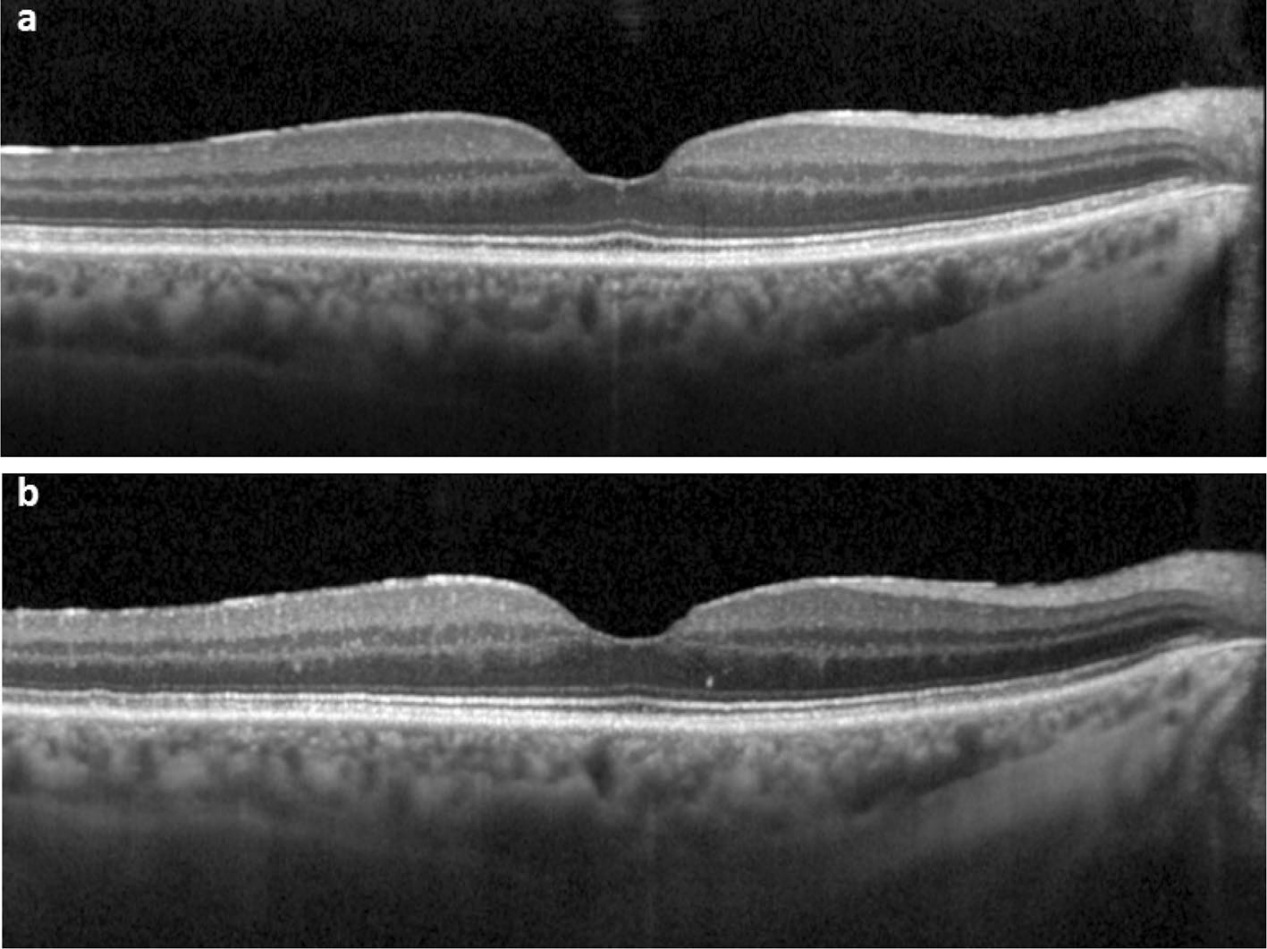
SD-OCT of the fovea (horizontal line scan) with the foveal crack sign at baseline without further progression in a 70-year-old-woman. (**a**) Mild foveal crack sign is present. (**b**) Resolution of the foveal crack sign 13 months later.

Progression rate was the highest in eyes with FCS and posterior vitreous adhesion (77%; 10 of 13 eyes), followed by the group without FCS with posterior vitreous adhesion (4%; 2 of 48 eyes). In both groups with posterior vitreous detachment, no cases of progression to FTMH formation have been observed, though FCS was present in 6 of 54 eyes. Figures 1, 2 and 3 show different disease courses over the observation period.

The predictive value of FCS regarding future FTMH formation was 52.6% (95% CI 29.5–74.8%) in the positive test and 97.9% (95% CI 91.8–99.6%) in the negative test. The sensitivity was 83.3% (95% CI 50.9–97.1%) and the specificity 91.1% (95% CI 83.3–95.6%).

The predictive value was higher, when FCS and vitreous adhesion were present simultaneously—76.9% (95% CI 46.0–93.8%) for the positive test and 98.0% (95% CI 92.4–99.7%) for the negative test. The sensitivity remained stable at 83.3% (95% CI 50.9–97.1%) and the specificity increased to 97.1% (95% CI 91.1–99.2%).

## Discussion

In the present study, we analyzed SD-OCT images of fellow eyes of patients with FTMH. In our cohort of patients, the novel previously described^8,9^ OCT finding termed “foveal crack sign” could be seen in 19 of 113 cases (17%). 10 (53%) of these 19 eyes developed a FTMH during the observation period. FCS seems to represent an additional risk factor—besides vitreomacular adhesion or traction—for development of FTMH. In a recent work of Ishibashi et al. FCS was shown to precede macular hole formation after vitrectomy in 10 eyes of 10 patients^8^. Scharf et al. showed in a recent study, that 50% of the eyes with FTMH displayed the foveal crack sign preceding macular hole formation^9^. In our study, 10 (83%) of 12 cases of FTMH development demonstrated FCS prior to FTMH formation. This higher rate might be due to the selection of patients. While Scharf et al. selected patients with FTMH in the first eye, we analyzed fellow eyes of patients with FTMH. Fellow eyes are known to have a higher risk of MH development^1–4^.

The exact mechanism of the appearance of this hyperreflective line is not known. Foveal crack sign can only be seen in the foveola, where a specialized population of Müller cells exists^10,11^. Bringmann et al. outlined, that these specialized Müller cells do not support neuronal activity but might serve optical and structural functions^10^. Tractional forces to the foveola in case of ERM or a vitreoretinal traction might lead to the disruption of the Müller cell cone with cell disorganization in the naturally existing cleavage plane. These structural changes in the fovea centralis might be captured on SD-OCT in the form of a hyperreflective vertical line in the umbo.

Because of the very small size of the umbo, it is not guaranteed to examine the umbo in standard OCT protocols. It might therefore be possible, that the “outer foveal defect” described by Choi et al.^6^ might represent an incomplete FCS. This hypothesis can be supported by the fact that all patients who showed this outer foveal defect in the study of Choi et al. developed a FTMH. This high MH development rate is similar to 77% of progression to FTMH formation in our study for patients with vitreous adhesion and FCS.

In the study of Ishibashi et al., all 10 FCS cases appeared with parafoveal ERM but no cases with ERM covering the foveola. The authors postulated, that in these eyes FCS might imply the dehiscence of the Müller cell cone caused by the parafoveal traction due to ERM. In our study, there were a few ERM cases in each group with no statistical significance across the different groups. All cases with further progression to FTMH formation were observed in both groups with posterior vitreous adhesion, while in the group with simultaneously observed FCS the progression rate was much higher (77% vs. 4%). Therefore, we suppose that the tractional forces of the posterior vitreous interface lead to the central foveal dehiscence and development of the foveal crack sign. This might indicate a special role of vertical traction in the development of this condition. This has already been suggested by Kumagai et al.^6^, who showed alterations of the vitreoretinal interface in *both* eyes of patients with only unilateral FTMH. The study of Choi et al.^7^ supports this furthermore by pointing out that patients without vitreofoveal adhesion had no substantial risk for FTMH development. The same result was found in our study population: there was no FTMH in fellow eyes within the vitreous detachment group. On the other hand, vitreofoveal adhesion cannot be the sole cause for development of FTMH, as only 12 out of 61 patients who showed this condition in our study progressed to FTMH.Regarding 9 cases with FCS at baseline, which did not progress to FTMH, it should be mentioned, that 5 of them resolved over the course of the study. In the 4 remaining cases, FCS was still present at the end of the study. Ishibashi et al. observed relatively late development of macular holes after FCS appearance with a mean time frame of 232 ± 171 days^7^. It might be possible, that these 4 eyes in our patients’ cohort could develop a macular hole later in the course of the disease.

The major limitation of this study is the retrospective nature. As the foveal crack sign is a very subtle structural change, its incidence might be underestimated on captured SD-OCT images with insufficient resolution and density of the scans. On the other hand, because of the subtle nature of this change its incidence might as well be overestimated, as it is still a subjective assessment. Investigating interobserver variability in identifying FCS—which was not the subject of our study—could bring up more information regarding its possible routine application in everyday practice. In our opinion, high resolution dense volume OCT scans are more suitable for detecting this finding. As these scan protocols are not routinely used in our clinic, they could not be included in this retrospective analysis.

One further limitation is a small study population of patients with FTMH and foveal crack sign in the fellow eye. The comparison across the four study groups should be regarded with caution because of the highly different sample size in the groups. Furthermore, the group with FCS and posterior vitreous adhesion showed statistically significant longer follow up time. We believe that the different values and the longer follow up were caused by choosing the last examination in the hospital as follow up time. This means that patients who developed a FTMH in the fellow eye were seen again in the hospital (= long follow up), while others that did not develop FTMH were not (= short follow up).

To the best of our knowledge, our study is the first to report the incidence of the foveal crack sign in fellow eyes of patients with FTMH. FCS showed a sensitivity of 83.3% and specificity of 91.1% in predicting macular hole formation over the study course of 21 months. In case of FCS and posterior vitreous adhesion, the specificity of predicting future macular hole development increased to 97.1%. We recommend, that eyes with FCS and attached posterior vitreous should be monitored closely to detect early stages of macular hole development. If the high incidence of FTMH formation in eyes with FCS and attached posterior vitreous (77%) can be supported in future prospective studies, the indication for an early vitrectomy with nowadays very low complication rate should be discussed for those cases.

## Methods

The present study was approved by the Institutional Review Board of Saxony (Dresden, Germany) under the number EK-BR-108/19-1 and adhered to the tenets of the Declaration of Helsinki. Informed patients’ consent was waived because of the retrospective anonymous design and because no study-related investigations were necessary.

### Patient selection

The patient database in Klinikum Chemnitz was reviewed for billing codes of macular hole according to the International Classification of Diseases, 10th Revision between January 2015 and December 2018. Patients included in this study met the following criteria: (1) diagnosed with FTMH in one eye; (2) spectral-domain optical coherence tomography (SD-OCT) at every visit with image quality score > 30 and the coverage of the umbo. The exclusion criteria were: (1) retinal or macular disease (e.g., high myopia, severe age-related macular degeneration), that confounded the analysis of the foveal structure in the fellow eye; (2) one-eyed patients; (3) patients with unclear status of posterior vitreous on SD-OCT images.

### Ophthalmic examination

All patients underwent a complete ophthalmic examination of both eyes including BCVA testing, applanation tonometry, slit-lamp biomicroscopy, indirect binocular ophthalmoscopy and SD-OCT imaging. SD-OCT examination was performed using Spectralis OCT (Heidelberg Engineering Inc., Heidelberg, Germany). The macula was scanned with an acquisition speed of 40,000 A-scans per second using “fast macular volume” protocol, consisting of a 25-line horizontal raster scan covering 20° × 20° centered on the fovea with standard nine frames. Additionally, the single horizontal foveal scan covering 20° was also analyzed for posterior vitreous status in the optic nerve head area. The eye tracking system (ART Module, Heidelberg Engineering Inc.) was used to minimize motion artifacts.

### SD-OCT image assessment

**Figure 4:**
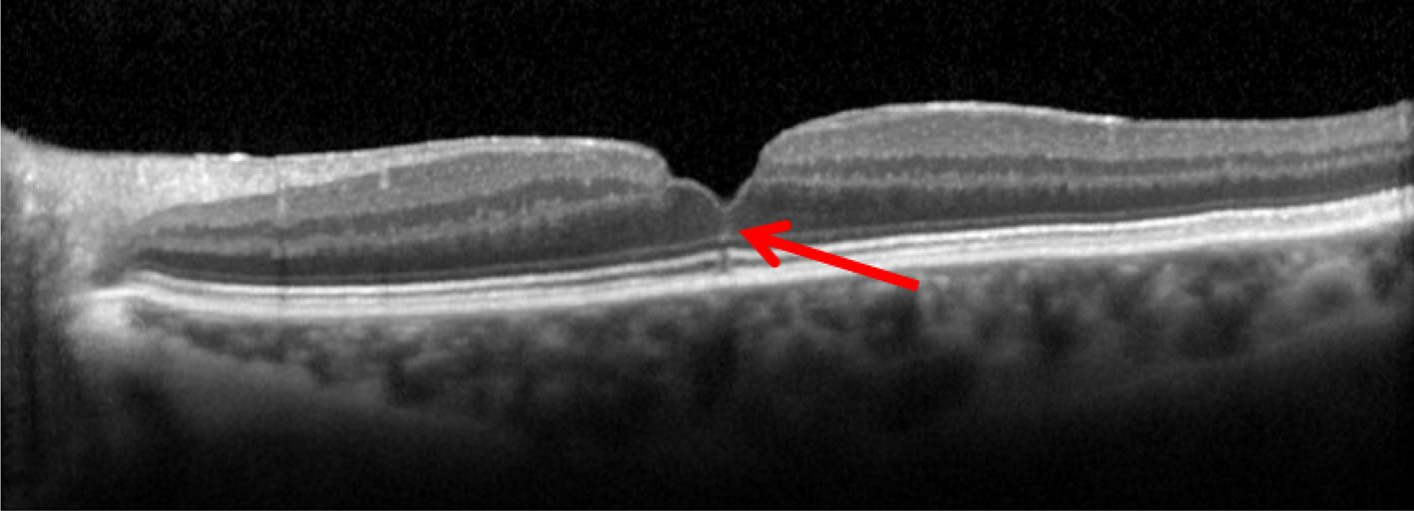
SD-OCT of the fovea (horizontal line scan) with the foveal crack sign defined as a vertical hyperreflective line in the umbo extending from the ellipsoid zone through middle retinal layers reaching the internal limiting membrane. Note the presence of a lamellar macular hole as well as a mild epiretinal membrane.

OCT volume and line scans were studied by one observer (O.F.) in all fellow eyes to determine the presence or absence of the foveal crack sign. The foveal crack sign was defined as a hyperreflective vertical line in the umbo extending from the ellipsoid zone through middle retinal layers reaching the internal limiting membrane (ILM) as previously defined by Scharf et al.^7^ The second observer (E.M.) determined the central vitreoretinal interface status on SD-OCT images. Figure 4 shows an example of a foveal crack sign.

According to SD-OCT images evaluation, patients were divided into 4 groups for further analysis: patients with FCS and vitreous adhesion, patients with FCS and vitreous detachment, patients without FCS with vitreous adhesion, patients without FCS with vitreous detachment. Vitreous adhesion was defined as visible attached posterior vitreous either across the whole scan area including the optic nerve head or only in the vitreomacular region. Eyes with vitreomacular traction were also analyzed in the group with vitreous adhesion.

### Statistical analysis

IBM SPSS Statistics, version 27.0.0.0 for Windows (IBM, Armonk, NY, USA) was used to perform the analysis. Visual acuity measurements were converted from decimal numbers to logMAR for all analyses. Data for continuous variables are expressed as mean ± standard deviation. One-way multivariate factorial ANOVA with post hoc Sidak’s test and the chi-square test according to the variables was performed to compare differences across the four groups. For representing statistical significance, *p* < 0.05 was chosen. The sensitivity and specificity for FCS predicting FTMH formation were calculated using Bayesian sensitivity analysis.

## References

[1] Gass JDM (1991). Risk of developing macular hole. Arch. Ophthalmol..

[2] Gass JDM, Joondeph BC (1990). Observations concerning patients with suspected impending macular holes. Am. J. Ophthalmol..

[3] Akiba J, Kakehashi A, Arzabe CW, Trempe CL (1992). Fellow eyes in idiopathic macular hole cases. Ophthalmic Surg..

[4] Lewis ML, Cohen SM, Smiddy WE, Gass JDM (1996). Bilaterality of idiopathic macular holes. Graefe's Arch. Clin. Exp. Ophthalmol..

[5] Ezra E (1998). Incidence of idiopathic full-thickness macular holes in fellow eyes: a 5-year prospective natural history study. Ophthalmology.

[6] Kumagai K (2011). Vitreoretinal Interface and foveal deformation in asymptomatic fellow eyes of patients with unilateral macular holes. Ophthalmology.

[7] Choi JH (2020). Development of idiopathic macular hole in fellow eyes: spectral domain optical coherence tomography features. Retina.

[8] Ishibashi T (2020). Foveal crack sign: an optical coherence tomography sign preceding macular hole after vitrectomy for rhegmatogenous retinal detachment. Am. J. Ophthalmol..

[9] Scharf JM (2020). Hyperreflective stress lines and macular holes. Invest. Ophthalmol. Vis. Sci..

[10] Bringmann A (2018). The primate fovea: structure, function and development. Prog. Retin Eye Res..

[11] Syrbe S (2018). Müller glial cells of the primate foveola: an electron microscopical study. Exp. Eye Res..

